# Cataloging metagenome-assembled genomes and microbial genes from the athlete gut microbiome

**DOI:** 10.20517/mrr.2023.69

**Published:** 2024-07-22

**Authors:** Laura Wosinska, Liam H. Walsh, Calum J. Walsh, Paul D. Cotter, Caitriona M. Guinane, Orla O’Sullivan

**Affiliations:** ^1^Department of Biological Sciences, Munster Technological University, Cork Campus, Cork T12 P928, Ireland.; ^2^Teagasc Food Research Centre, Moorepark, Fermoy, Cork P61 C996, Ireland.; ^3^APC Microbiome Ireland, Cork T12 YT20, Ireland.; ^4^VistaMilk, Fermoy, Cork P61 C996, Ireland.; ^#^Authors contributed equally.

**Keywords:** Athlete, microbiome, MAGs, genes, novel species, *in silico*

## Abstract

**Aim:** Exercise has been increasingly recognized as a potential influencer of the gut microbiome. Nevertheless, findings remain incongruous, particularly in relation to sport-specific patterns.

**Methods:** In this study, we harness all publicly available data from athlete gut microbiome shotgun studies to explore how exercise may influence the gut microbiota through metagenomic assembly supplemented with short read-based taxonomic profiling. Through this analysis, we provide insights into exercise-associated taxa and genes, including the identification and annotation of putative novel species from the analysis of approximately 2,000 metagenome-assembled genomes (MAGs), classified as high-quality (HQ) MAGs and assembled as part of this investigation.

**Results:** Our metagenomic analysis unveiled potential athlete-associated microbiome patterns at both the phylum and species levels, along with their associated microbial genes, across a diverse array of sports and individuals. Specifically, we identified 76 species linked to exercise, with a notable prevalence of the *Firmicutes* phylum. Furthermore, our analysis detected MAGs representing potential novel species across various phyla, including *Bacteroidota*, *Candidatus Melainabacteria*, *Elusimicrobia*, *Firmicutes*, *Lentisphaerae*, *Proteobacteria*, *Tenericutes*, and *Verrucomicrobiota*.

**Conclusion:** In summary, this catalog of MAGs and their corresponding genes stands as the most extensive collection yet compiled from athletes. Our analysis has discerned patterns in genes associated with exercise. This underscores the value of employing shotgun metagenomics, specifically a MAG recovery strategy, for pinpointing sport-associated microbiome signatures. Furthermore, the identification of novel MAGs holds promise for developing probiotics and deepening our comprehension of the intricate interplay between fitness and the microbiome.

## INTRODUCTION

Gut microbes are significant drivers of human health and disease. They perform vital functions such as breaking down indigestible food components, synthesizing essential vitamins, safeguarding against pathogens, and modulating the immune system^[1-5]^. Factors such as birth mode, health conditions, dietary habits, and medication use can significantly shape the development and stability of the microbiome^[6]^. Several investigations have explored the relationship between fitness levels and microbial diversity, revealing that athletes tend to harbor a more diverse microbiome compared to non-athletes^[7-9]^. Regular physical activity has long been associated with numerous health benefits, including improved cardiovascular function^[7]^, enhanced mental well-being^[8]^, and reduced incidence of illness^[9]^. Recently, attention has turned to the potential impact of exercise, specifically physical fitness, on the gut microbiome^[10-12]^. For example, specific bacterial taxa, such as *Akkermansia*^[10,11,13-15]^, *Coprococcus*^[15]^, *Faecalibacterium*^[15-17]^, *Prevotella*^[14]^, *Ruminococcus*^[14,15,17]^, *Roseburia*^[15,16]^, and *Veillonella*^[12]^, have been found to be more abundant in athlete populations. Moreover, emerging evidence suggests that this modulation of microbial diversity correlates with the production of microbial metabolites that can confer beneficial effects on human physical performance and overall health^[18]^. Additionally, O’Donovan *et al.* highlighted notable taxonomic distinctions among various sports disciplines, suggesting a unique microbiome signature for each sport^[19]^. Despite the absence of specific recommendations for microbiome-modifying interventions in athletes, the growing interest underscores the untapped potential of the gut microbiome in optimizing athletic performance. Moreover, while some studies suggest that exercise may correlate with an increase in certain beneficial bacteria, others indicate that intense exercise could potentially lead to a decrease in microbial diversity or alterations in specific taxa. For instance, high-intensity exercise has been associated with reduced abundance of certain taxa, such as *Bacteroides*, typically linked to a healthy gut microbiome. However, the extent and significance of these changes may vary based on factors such as exercise type, duration, and intensity, as well as individual differences in diet, genetics, and gut microbiome composition^[20]^. Therefore, while exercise may exert both positive and negative effects on specific taxa within the gut microbiome, further research is essential to fully comprehend the complexities of this relationship. Our work aims to further explore the intricate microbiome-fitness paradigm through an *in silico* approach of publicly available metagenomics datasets (*n* = 7)^[11,12,14,19,21,22]^, obtained from fecal samples of Athletes (nMALE = 335, nFEMALE = 180) involved in 19 different sports types and Control populations of individuals deemed to adhere to a sedentary lifestyle (low physical activity) (nMALE = 106, nFEMALE = 61). Specifically, we apply a metagenome-assembled genome (MAG) approach to enable an in-depth identification of functional features within athlete-associated microbial genomes, extending to putative new species^[21]^. Ultimately, our work highlights the potential of culture-independent techniques, namely a MAG recovery strategy, to expand our understanding of the specific roles that the microbes and their genes play in fitness.

## METHODS

### Metagenomic sample collection

For this study, we considered all publicly available metagenomic studies (*N* = 5) of different sport types^[11,12,14,19,21]^ and one unpublished study PRJEB46398 (Runners) from our laboratory. The accession numbers for the studies used are PRJEB15388 (Rugby players)^[11]^, PRJNA472785 (Boston marathon runners)^[12]^, PRJNA305507 (Cyclists)^[14]^, PRJEB32794 (The Irish Olympic team)^[19]^, and PRJEB28338 (Cricketers)^[21]^ along with controls (non-athletes) that were included as control groups in some of the studies or derived from PRJEB20054^[22]^.

### Metagenomic data processing and taxonomic profiling

All metagenomic processing was conducted using the Teagasc high-performance computing cluster. Initially, all paired-end reads were obtained from NCBI and subjected to quality control (QC) procedures through a validated pipeline (available at https://github.com/SegataLab/preprocessing), consisting of three subsequent steps: (i) discarding of low-quality (quality < 20), short (L < 75 bp) and with too many ambiguous nucleotides (*N* > 2) reads using Trim Galore v0.6.6 (https://www.bioinformatics.babraham.ac.uk/projects/trim_galore/); (ii) removal of human (HG19 human genome release) and bacteriophage phiX174 DNA (Illumina spike-in) contamination by mapping reads against their reference genomes through BowTie2 v2.2.9 (with parameter –sensitive-local)^[23,24]^; (iii) Reads passing the filters were then sorted and split reads into forward, reverse, and unpaired files. Cleaned-up reads were used for the subsequent de novo metagenome assembly and short read-based and taxonomic profiling. Taxonomic read mapping-based profiles were generated using MetaPhlAn 4 v4.0.6 using the MetaPhlAn 4 database v0.6. The scripts for the bioinformatics tools described above were generated utilizing the resources available at the GitHub repository: https://github.com/SegataLab/MASTER-WP5-pipelines. These scripts were derived and adapted from the repository’s collection.

### Extraction of MAGs and their taxonomic assignment

Metagenomic assemblies were generated using pipeline proposed and validated in^[25]^ based on subsequent steps: (i) metagenomic assembly with MEGAHIT v1.1.3; (ii) removal of contigs shorter than 1,000 bp; (iii) alignment of the remaining contigs against original raw data with Bowtie2 v2.2.9121 to calculate coverage information; (iv) binning of the contigs through MetaBAT v2.12.1123; (v) QC of the resulting putative genomes with CheckM2 v0.1.3^[26]^ and successive filtering of high-quality (HQ) (completeness > 90% and contamination < 5%) and medium-quality (MQ) (completeness ≥ 50% and contamination < 5%) MAGs as established by Bowers *et al.*^[27]^. All HQ MAGs were advanced for further analysis. The recovered MAGs for all control metagenomes derived from participants with a sedentary lifestyle were pooled into one group representing the sedentary control population. Prokka v.1.13^[28]^ was used to functionally annotate HQ MAGs. Taxonomy of recovered HQ MAGs and identification of potentially novel species were determined using PhyloPhlan3 v.3.0.2^[29]^, with the SGB Jun23 database and a default assignment threshold of 5%. The phylogenetic tree of life was constructed using PhyloPhlAn, incorporating 400 universal markers provided by PhyloPhlAn. Parameters were configured as follows: “--diversity high --fast --min_num_markers 50”. dRep v 3.2.0^[30]^ was used to cluster MAGs representing putative new species into primary and secondary clusters based on their relative similarities, using the Average Nucleotide Identity (ANI) as a measure. The default threshold for forming primary (MASH) clusters was set at 0.9, while the default threshold for forming secondary clusters is set at 0.99. The relative abundance, expressed as Reads per Kilobase per Million Mapped Reads (RPKM) of MAGs, was computed by mapping metagenomic reads using CoverM v0.6.1 (https://github.com/wwood/CoverM).

### Establishing a microbial gene catalog

A gene catalog was established using HQ MAGs. HQ MAGs were annotated using Prokka. The output (.gff format) was used to assess gene presence and absence using Roary^[31]^. The gene presence/absence file from Roary was used to analyze the difference in accessory genes between sedentary control and athlete populations. To assess gene presence/absence and their associations with specific traits, the gene presence/absence output from Roary, along with traits of interest file, was used as input to Scoary^[32]^. Scoary then scored the genes based on their associations with traits - in our case, exercise status. Associations were calculated using pairwise comparison algorithms, which calculate the maximum and minimum number of pairs that support an association. A Bonferonni adjusted *P* value of < 0.05 was considered significant. An odds ratio of < 1 indicated a negative association, while an odds ratio of > 1 indicated a positive association.

### Statistical analysis and data visualization

All statistical analysis was performed in R (v4.1.2) implemented using R studio. Community-level microbiome analysis was carried out with hillR (v0.5.2)^[33]^ to compute alpha and beta diversity values. The stats (v4.1.2) kruskal.test function was used to perform the Kruskal-Wallis rank sum test to identify significant differences in alpha diversity values. The adonis function in the vegan package^[34]^ was used for the permutational analysis of variance (PERMANOVA)^[35]^. The pairwiseAdonis function in the pairwiseAdonis (v0.4)^[36]^ package was used to perform pairwise comparisons of Adonis using Benjamini-Hochberg correction for multiple comparisons. The clustering of samples was visualized by non-metric multidimensional scaling (NMDS) using vegan’s metaMDS function, and the relationship between microbiome composition and sample metadata (gender, sport, *etc.*) was investigated using vegan’s (v2.6.2)^[34]^. The envfit function performs multiple regression of the metadata and covariates with the NMDS ordination axes and generates a *P*-value through permutation testing. The statistical package Microbiome Multivariable Associations with Linear Models (MaAsLin)2^[37]^ was used to find associations between sample metadata and bacterial abundance. Data visualization of summary statistics generated from the above tests was performed using the ggplot2 package^[38]^.

## RESULTS

### Exploration and meta-analysis of athlete-associated metagenomic data

**Figure 1:**
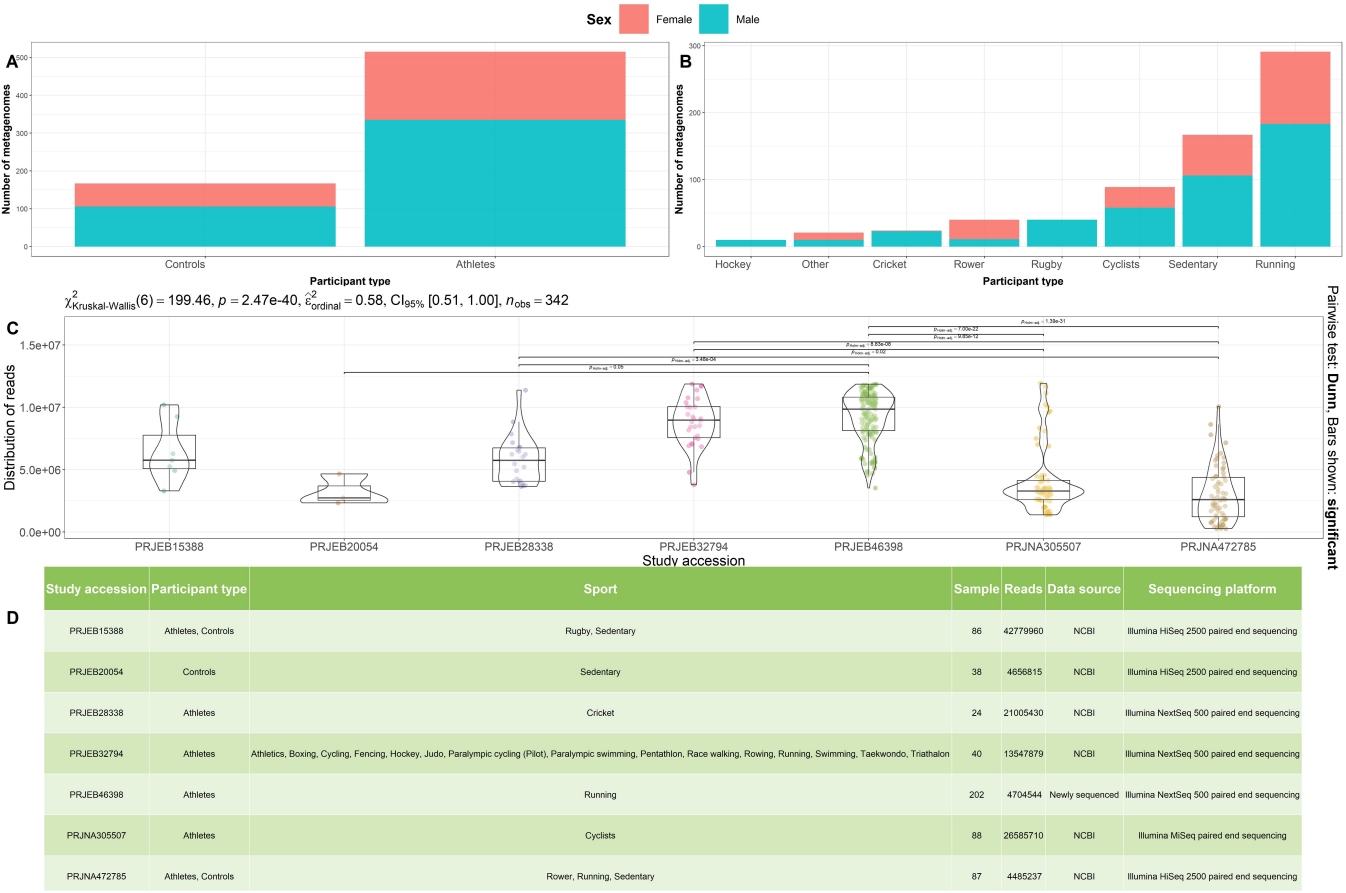
Cohort overview, including sample and metadata exploration. (A) Number of metagenomes (*Y*-axis) separated derived from athlete and sedentary control populations; bar charts are colored according to the variables “Female” (Red) and “Male” (Blue); (B) Number of metagenomes (*Y*-axis) separated according to exercise group (*X*-axis); bar charts are colored according to the variables “Female” (Red) and “Male” (Blue); (C) The 25th to 75th percentile distribution of reads (*Y*-axis) across the datasets considered in this study (*X*-axis); (D) Summary description of each metagenomics dataset considered in this study; details include ENA study accession number, metadata of the metagenomes, number of metagenomes, average number of reads per dataset, and sequencing platform used. ENA: European Nucleotide Archive.

To further investigate the potential link between physical activity and gut microbiota composition, we compiled a comprehensive dataset comprising 682 shotgun metagenomics datasets generated using Illumina sequencing technology and publically available in the NCBI repository [Supplementary Table 1]. Metagenomics datasets were derived from fecal samples provided by different athlete populations (nMALE = 335, nFEMALE = 180) [Figure 1A] or control populations of healthy adults with sedentary lifestyles (nMALE = 106, nFEMALE = 61) [Figure 1B]. Athlete populations represented moderate, recreation, and elite athletes from 19 different sports types, and included Runners^[12]^ (nMALE = 183, nFEMALE = 108), Cyclists^[14]^ (nMALE = 58, nFEMALE = 31), Rowers^[12]^ (nMALE = 11, nFEMALE = 29), Rugby players^[11]^ (nMALE = 40, nFEMALE = 0), and Cricket players^[24]^ (nMALE = 23, nFEMALE = 1) [Figure 1]. Each sample was accompanied by categorical metadata detailing the participant’s gender and physical activity levels. Following QC and filtering steps. It is important to note the statistical differences in the number of reads between a number of the metagenomics datasets considered in this study (*P* ≤ 0.05) (Kruskal-Wallis multiple comparisons) [Figure 1C]. The average number of reads ranged from 4,485,237 (PRJNA472785) to 42,779,960 (PRJEB15388) [Figure 1D].

### Short-read taxonomic profiling reveals associations at the phylum and species level based on general exercise

Initially, we utilized short-read taxonomic profiling to offer a comprehensive view of microbial diversity, encompassing the distribution and abundance of microbial taxa across various athlete groups and the sedentary control cohort. We first identified prevalent species based on the following criteria: a relative abundance of ≥ 0.1% in ≥ 10% of samples, as defined for this study. In the athlete dataset, a total of 188 prevalent species were detected, distributed across the phyla *Actinobacteria* (5), *Bacteroidetes* (30), *Euryarchaeota* (1), *Firmicutes* (146), *Planctomycetes* (1), *Proteobacteria* (4), and *Verrucomicrobia* (1). Similarly, in the control sedentary population, a total of 186 prevalent species were detected, encompassing the phyla *Actinobacteria* (5), *Bacteroidetes* (40), *Candidatus Melainabacteria* (1), *Euryarchaeota* (1), *Firmicutes* (126), *Planctomycetes* (1), *Proteobacteria* (11), and *Verrucomicrobia* (1).

**Figure 2:**
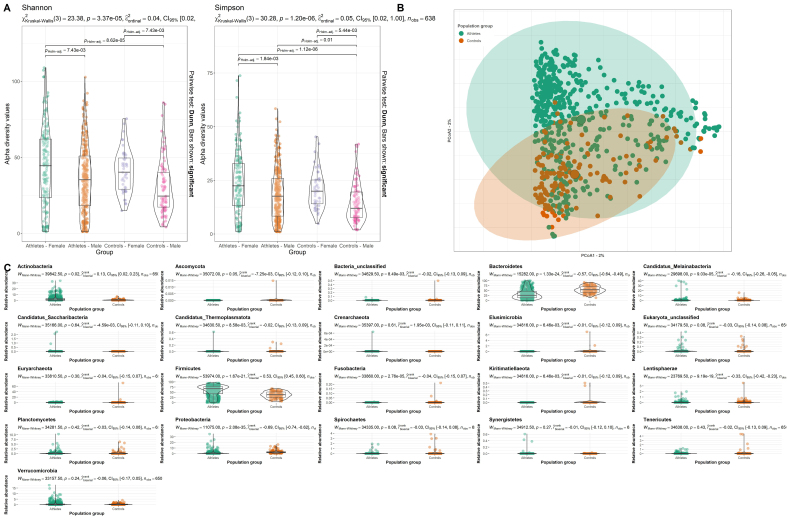
Influence of exercise on microbiome diversity. (A) Differences in alpha diversity between the variables “Athletes - Female” (Green), “Athletes - Male” (Orange), “Controls - Female” (Purple), and “Controls - Male” (Pink) values calculated using the Shannon and Simpson diversity metrics; (B) PCoA of beta diversity by intervention, measured by Bray-Curtis dissimilarity and calculated for species-level composition. Points are colored according to the variables “Athletes” (Green) and “Controls” (Orange); (C) Significant changes in the relative abundance of certain phylum across the population groups “Athletes” (Green) and “Controls” (Orange). The bounds, whiskers, and percentile of each box plot represented maximum, 75th percentile, median, 25th percentile, and minimum from the top to the bottom, respectively. PCoA: Principal coordinate analysis.

Evaluation of within-sample diversity using both the Simpson Index and Shannon Index alpha diversity metrics revealed no significant differences between the athlete and sedentary populations (Kruskal-Wallis *P* > 0.005) [Figure 2A]. Further separation of the populations by sex revealed no significant differences for either index, but we noted consistently higher alpha diversity within female populations compared to male populations including Athletes - Female *vs.* Athletes - Male (Kruskal-Wallis *P* < 0.01), Athletes - Female *vs.* Controls - Male (Kruskal-Wallis *P* < 0.01), and Controls - Female *vs.* Controls - Male (Kruskal-Wallis *P* > 0.01) [Figure 2A].

Evaluation of microbial diversity between different metagenomes using the Bray-Curtis dissimilarity index (Adonis analysis, r^2^ = 0.05, *P* < 0.01), modeling with envfit (envfit analysis, r^2^ = 0.1281, *P* < 0.01) and MDS [Figure 2B] all revealed a perceivable shift in the composition of the microbiome according to population group. While *Firmicutes* and *Bacteroidota* were the most abundant and prevalent phyla among all metagenomes considered in this study [Figure 2A], we observed statistically significant increases in the relative abundance of *Actinobacteria* (Rank biserial = 0.13, *P* = 0.02) and *Firmicutes* (Rank biserial = 0.53, *P* < 0.01) within the athletes population compared to the control population [Figure 2C]. In contrast, when considering the control populations, we observed statistically significant increases in the relative abundance of *Ascomycota* (Rank biserial = 0.00735, *P* < 0.01), *Bacteria unclassified* (Rank biserial = 0.02, *P* < 0.01), *Bacteroidetes* (Rank biserial = 0.57, *P* < 0.01), *Candidatus Melainabacteria* (Rank biserial = 0.16, *P* < 0.05), *Candidatus Thermoplasmatota* (Rank biserial = 0.02, *P* < 0.01), *Elusimicrobia* (Rank biserial = 0.01, *P* < 0.01), *Fusobacteria* (Rank biserial = 0.04, *P* < 0.05), *Kiritimatiellaeota* (Rank biserial = 0.01, *P* < 0.01), *Lentisphaerae* (Rank biserial = 0.33, *P* < 0.01) and *Proteobacteria* (Rank biserial = 0.69, *P* < 0.01) [Figure 2C].

Previous research has identified several genera that are often elevated in athletes, including *Akkermansia* (10), *Alistipes* (19), *Eubacterium* (19), *Faecalibacterium* (15), *Prevotella* (14), *Ruminococcus* (15), *Roseburia* (15), and *Collinsella*^[39]^. To corroborate these findings, we analyzed the relative abundance of these genera across sedentary control populations and athletes. Our analysis revealed statistically significant increases in the relative abundance of *Alistipes* (Rank biserial = 0.51, *P* < 0.01), *Faecalibacterium* (Rank biserial = 0.22, *P* < 0.01), *Roseburia* (Rank biserial = 0.13, *P* = 0.01), and *Ruminococcus* (Rank biserial = 0.26, *P* < 0.01) within the athletes population compared to the control population [Supplementary Figure 1].

In our analysis, we further identified 76 species associated with athletes, primarily from the phylum *Firmicutes* (72), with additional representation from *Actinobacteria* (2) and *Bacteroidetes* (2) [Supplementary Table 2] using the statistical package MaAsLin2. To ensure robustness, only genera with a *q* value < 0.2 were considered for this analysis. We further observed differential abundances of species within athletes across the genera often elevated in athletes, except *Prevotella.* Of these species, it was further noted that large majorities of these differentially abundant species belong to the following genera: *Clostridia unclassified* (14), *Alistipes* (11), and *Bacteroides* (11).

### Short-read taxonomic profiling reveals few sports-specific associations at the species level

**Figure 3:**
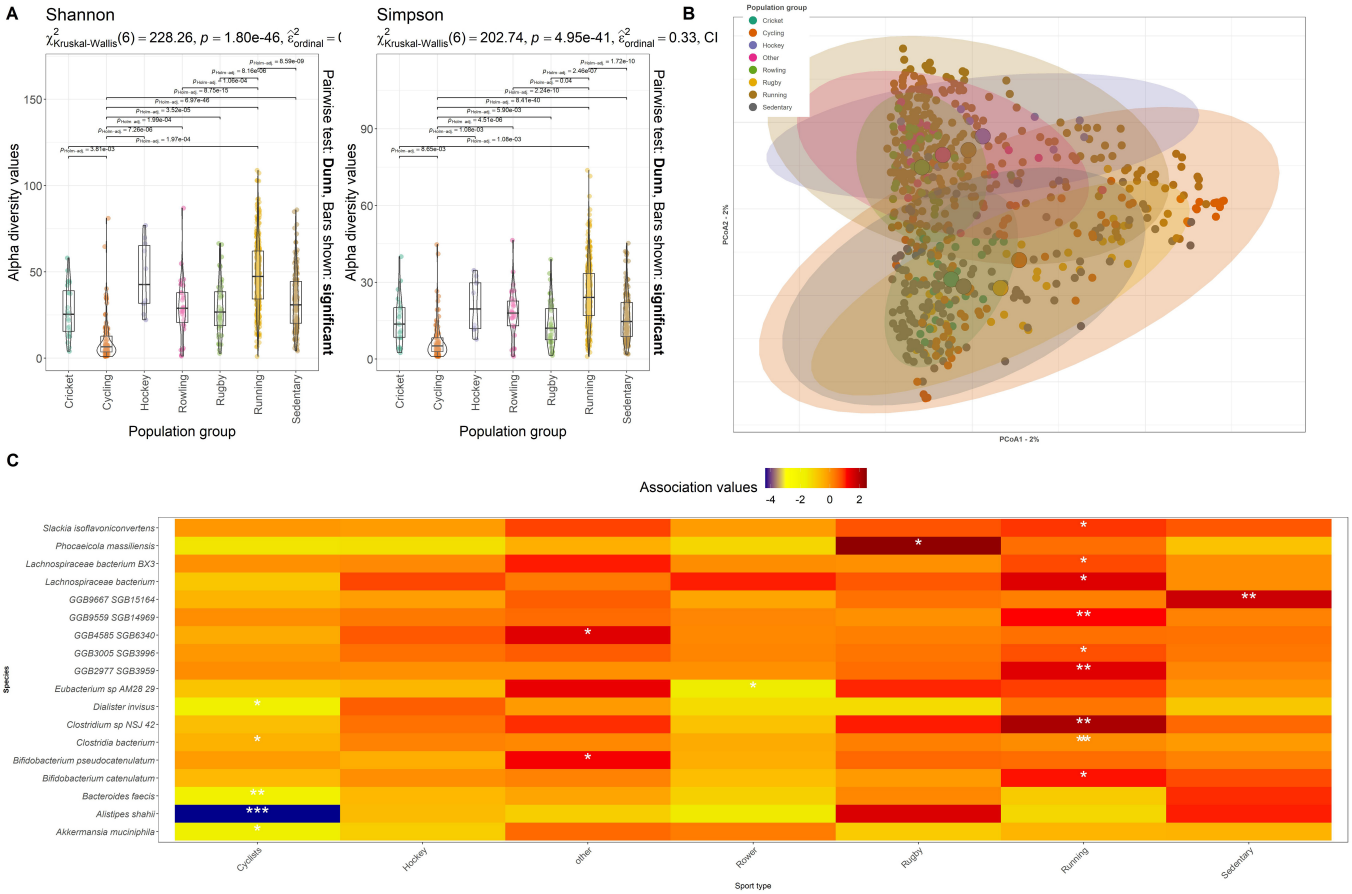
Influence of sport types on microbiome diversity. (A) Differences in alpha diversity calculated using the Shannon and Simpson diversity metrics across sports groups and sedentary controls; (B) PCoA of beta diversity by intervention, measured by Bray-Curtis dissimilarity and calculated for species-level composition. Points are colored according to sports and sedentary control populations; (C) Unique association scores as measured by MaAsLin2 between microbial species (*Y*-axis) and the population groups (*X*-axis). *q* values indicated as ^*^q < 0.2; ^**^q < 0.05; ^***^q < 0.001. PCoA: Principal coordinate analysis.

We examined microbial diversity across the various athlete types (Rowing, Rugby, Running, *etc.*) and the sedentary control population. Within-sample diversity using both the Simpson Index and Shannon Index alpha diversity metrics revealed significant differences in alpha diversity across the various sub-populations, with cyclists having consistently lower alpha diversity compared to all other groups including the sedentary control population (Dunn test, *P* < 0.05) [Figure 3A]. No specific sports population displayed consistently higher alpha diversity compared to all other groups (Dunn test, *P* > 0.05) [Figure 3A]. While significant differences were observed across certain athlete and sedentary populations (Dunn test, *P* < 0.05 and envfit analysis, r^2^ = 0.3611, *P* < 0.01), no distinct clustering was observed for any of the sub-populations based on MDS analysis [Figure 3B] and statistical testing using pairwise Dunn tests. However, significant associations between the relative abundance of species and specific sports were identified between the *q*-value range of < 0.01 and < 0.25. Statistically significant positive associations were found in Cyclists (*n* = 2), Hockey (*n* = 134), Rower (*n* = 33), Rugby (*n* = 152), and Running (*n* = 193). Statistically significant negative associations were found in Cyclists (*n* = 152), Hockey (*n* = 34), Rower (*n* = 105), Rugby (*n* = 38), and Running (*n* = 58). As expected, given the abundance and prevalence of the *Firmicutes*, *Bacteroidota* and *Actinobacteria* phyla among all metagenomes considered in this study, most of the associations were observed within these phyla. Of the associations reported, only 21 were sport-specific [Figure 3C]. Diversity was further assessed using a hierarchical clustering analysis (HCL) with bootstrap resampling to assess the stability of the clustering solution across multiple iterations. The optimal number of clusters was determined based on the stability of cluster assignments. HCL revealed four taxonomic clusters, distinctly separated (Dunn test, *P* < 0.05), with no exclusivity to exercise or sport. Proportional analysis of athletes and controls within clusters indicated significant differences in Clusters 1 and 3 (Pearson’s Chi-squared test *P* < 0.001). Notably, Cluster 1 comprised 50% of athlete metagenomes but only 8% of control sedentary metagenomes, while Cluster 3 consisted of 36% of control sedentary metagenomes compared to 16% of athlete metagenomes.

### MAGs and taxonomy assignment

**Figure 4:**
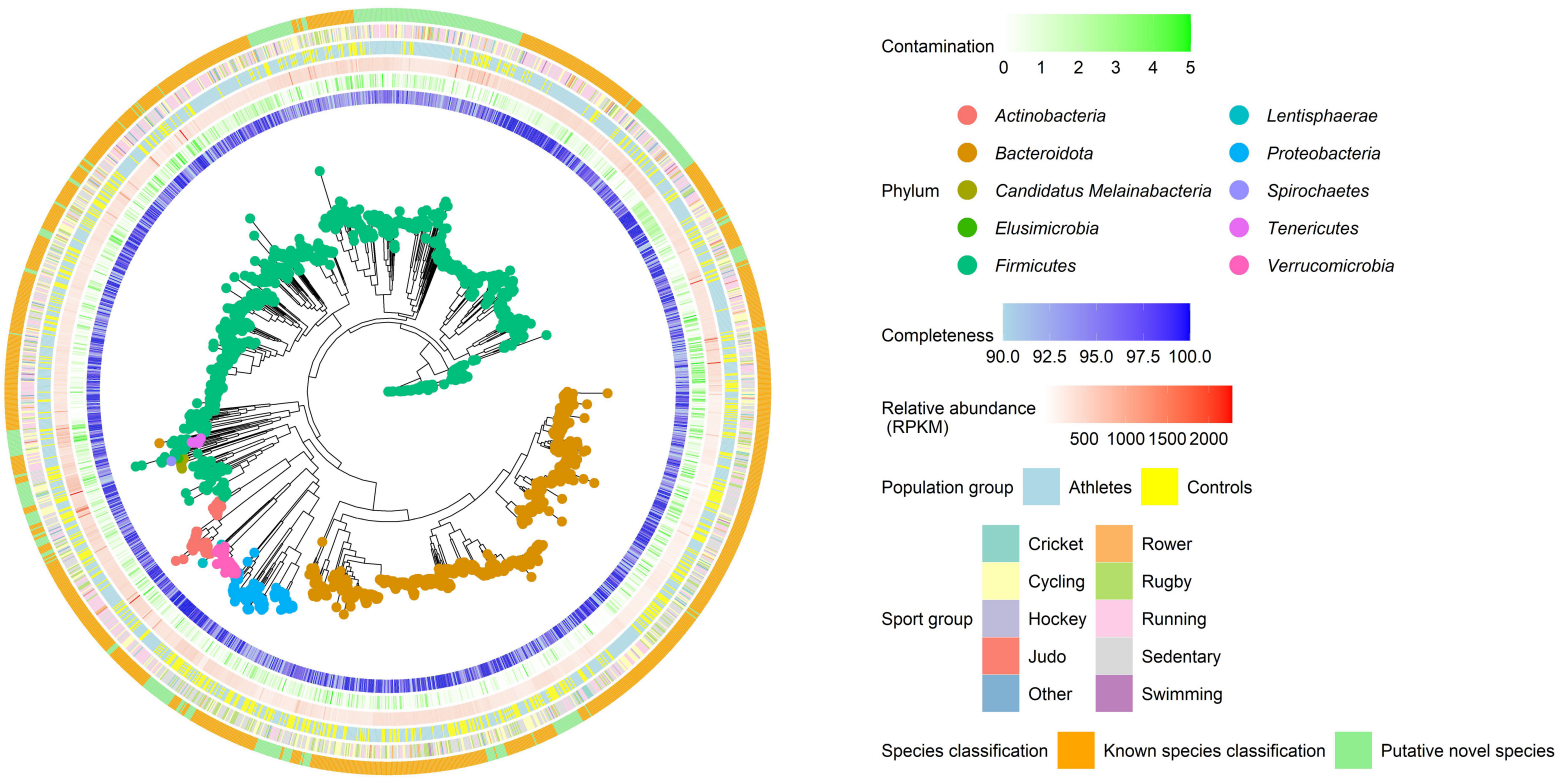
Phylogenetic Tree of HQ MAGs assembled in this study. This phylogenetic tree illustrates the differences in microbiome composition between athletes and controls, as well as among different sport types. The colored tips of the phylogram correspond to the phylum classification of the MAGs, as assessed using PhyloPhlan3. The first and second outer bands, colored blue and green, represent the % completion and contamination, respectively, as assessed using CheckM. The third outer band, colored red, represents the relative abundance (expressed in RPKM) of the MAG within the metagenome as assessed using CoverM. The fourth outer band represents the population group classification of the metagenome in which the MAG was recovered. Population group classifications include “Athletes” (Blue) and “Controls” (Yellow). The fifth outer band represents the specific sport group classification of the metagenome in which the MAG was recovered. The sixth outer band indicates which MAGs are deemed as “Known species classification” (Yellow) or “Putative novel species” (Green) as assessed using PhyloPhlan3. HQ: High-quality; MAGs: metagenome-assembled genomes.

In total, the assembly of the associated metagenomics reads analyzed in this study yielded 8,679 MAGs of various quality (High, Medium and Low). MAG quality was assigned in this work using a previously established threshold by Bowers *et al.*^[27]^. Of the total number of MAGs detected, 2,003 were considered HQ MAGs (≥ 90% Complete and ≤ 5% Contaminated). Of the HQ MAGs, 676 MAGs were recovered from sedentary controls and 1,327 were recovered from athlete populations. Only HQ MAGs were brought forward for further analysis and underwent taxonomy assignment using PhyloPhlan3 [Figure 4].

### MAGs classification and recovery provide further evidence into associations at the phylum and species level based on general exercise

Distinct differences were observed between athletes and controls at the species level regarding MAG recovery [Figure 4]. Initially, dRep was employed to assess the similarity or dissimilarity in MAG recovery between the metagenomic datasets of athletes and controls, focusing on the differences in the presence or absence of secondary clusters. These secondary clusters were determined based on a MAG grouping using a 99% ANI threshold. Disparities in the presence or absence of secondary clusters were noted between both population groups. Specifically, the athlete metagenomics dataset contained 791 secondary clusters, while the control sedentary population had 506 secondary clusters. Despite the substantial number of secondary clusters in both populations, only 96 secondary clusters were identified concurrently in both metagenomics datasets.

Consistent with our short-read taxonomic profiling findings, a majority of HQ MAG recoveries fell under the *Firmicutes* and *Bacteroidota* phyla [Figure 4]. Notably, we observed elevated recovery rates for the *Actinobacteria* (nATHLETE = 43, nSEDENTARY = 10) and *Firmicutes* (nATHLETE = 838, nSEDENTARY = 322) phyla within the athlete population compared to the control sedentary group [Figure 4]. Proportional analysis of MAG recoveries across athletes and control sedentary populations further indicated significant differences in the recovery of *Firmicutes* within the athlete population compared to the control sedentary group (Pearson’s Chi-squared test *P* < 0.001) [Figure 4]. Conversely, in the control sedentary population, we noted increased recovery rates for the *Proteobacteria* (nATHLETE = 38, nSEDENTARY = 53) and *Bacteroidota* (nATHLETE = 366, nSEDENTARY = 271) phyla compared to athletes, despite the smaller sample size representing the control sedentary group [Figure 4]. Proportional analysis of MAG recoveries further indicated significant differences in the recovery of *Proteobacteria* and *Bacteroidota* within the control sedentary group compared to the athlete population (Pearson’s Chi-squared test *P* < 0.001).

**Figure 5:**
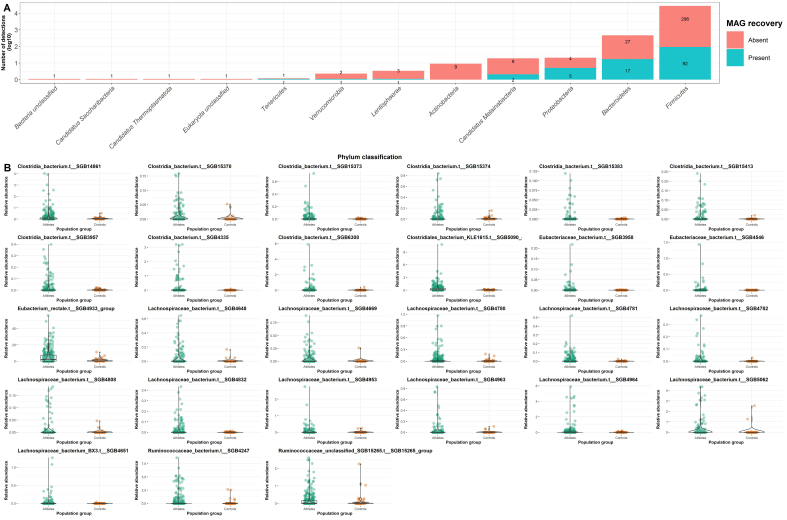
Number of detections classified as potentially novel species detected within this study. (A) Log10 representation of the number of unclassified detections (*Y*-axis) categorized by their respective phyla (*X*-axis). The bars are colored according to the variables “Absent” (Red) and “Present” (Blue), denoting whether a MAG was recovered with the same classification. White text labels indicate the frequency of detections and are arranged based on the variables “Absent” and “Present”; (B) Relative abundance of unclassified detections associated with the athlete population at the genus levels across “Athletes” (Green) and “Controls” (Orange) populations. The bounds, whiskers, and percentile of each box plot represented maximum, 75th percentile, median, 25th percentile, and minimum from the top to the bottom, respectively. MAG: Metagenome-assembled genome.

When analyzing the recovery rates of the seven bacterial genera often found to be increased in athlete populations, we noted heightened recovery rates for the following genera: *Akkermansia* (nATHLETE = 29, nSEDENTARY = 7), *Alistipes* (nATHLETE = 94, nSEDENTARY = 67), *Collinsella* (nATHLETE = 14, nSEDENTARY = 2), *Prevotella* (nATHLETE = 62, nSEDENTARY = 20), *Ruminococcus* (nATHLETE = 118, nSEDENTARY = 21), and *Roseburia* (nATHLETE = 34, nSEDENTARY = 23). However, proportional analysis of MAG recoveries only revealed significant differences in the recovery of *Alistipes* and *Ruminococcus* within the athlete population compared to the control sedentary group (Pearson’s Chi-squared test *P* < 0.001).

### The athlete gut as a storehouse for potential novel species

Further analysis was performed, leveraging both the short read and MAG results to identify potentially novel species associated with exercise. It is important to note that both athlete and control sedentary populations contain potentially novel species [Figure 4], with sedentary populations exhibiting statistically significant increases in the relative abundance of Bacteria unclassified (Rank biserial = 0.02, *P* < 0.01) compared to the athlete population [Figure 2C]. Overall, 984 species-level detections were observed using MetaPhlAn 4; of these detections, 474 were unclassified at the species level or higher. Most of the associations were observed within the *Firmicutes* and *Bacteroidota* phylum [Figure 5A]. Similarly, using a metagenomics assembly-based approach, we recovered 500 HQ MAGs that were unclassified at the species level or higher, belonging to the following phylum: *Bacteroidota* (75), *Candidatus Melainabacteria* (10), *Elusimicrobia* (1), *Firmicutes* (360), *Lentisphaerae* (1), *Proteobacteria* (38), *Tenericutes* (7) and *Verrucomicrobiota* (3) [Figure 5A].

Of the total unclassified detections accumulated, we further considered detections of the genera previously indicated to be increased in athletes according to the literature [e.g., *Akkermansia* (10), *Alistipes* (19), *Eubacterium* (19), *Faecalibacterium* (15), *Prevotella* (14), *Ruminococcus* (15), *Roseburia* (15), and *Collinsella*]^[39]^. In addition, we utilized MaAsLin2 for a more targeted search. Of the 76 differential abundant detections associated with the athlete population, 27 were unclassified at the species level or higher, including *Clostridia* unclassified (9), *Clostridiales* unclassified (1), *Eubacteriaceae* unclassified (2), *Lachnospiraceae* unclassified (13) and *Ruminococcaceae* unclassified (2) [Figure 5B and Supplementary Table 2]. Notably, all the differential abundant unclassified detections fell within the phylum *Firmicutes* and the class *Clostridia.* Within the athlete metagenomics dataset, we recovered HQ MAGs corresponding to 9 out of 27 unclassified differentially abundant detections linked to the athlete population. We further identified 9 novel MAGs within the family *Clostridiaceae* and a further 10 novel MAGs from the following genera of interest: *Alistipes* (5), *Faecalibacterium* (1), *Prevotella* (1), *Ruminococcus* (3). We have encountered repeated MAG recovery for many of the novel species recovered, including those potentially belonging to the *Clostridiaceae* family, as well as the *Ruminococcus* and *Alistipes* genera.

### Microbial gene catalog

A microbial gene catalog comprising 970,149 gene clusters was established using Roary^[31]^ software, as described in the materials and methods. Scoary^[32]^ was employed to establish associations between the presence or absence of gene clusters and general exercise. Overall, we identified 9,296 gene clusters, with 19,743 showing positive associations and 9,296 showing negative associations with exercise status. Among these gene clusters, the majority of annotations were attributed to hypothetical proteins, with 4,525 and 9,111 showing negative and positive associations with exercise status, respectively. Notably, athletes exhibited a prevalence of genes associated with stress adaptation, muscle recovery, and amino acid synthesis compared to the control sedentary control. Of the positively associated gene clusters with athletes, the most numerous annotations following hypothetical proteins included Adaptive-response sensory-kinase SasA (*n* = 71), Multidrug export protein MepA (*n* = 70), Vitamin B12 import ATP-binding protein BtuD (*n* = 66), putative ABC transporter ATP-binding protein (*n* = 61), putative protein (*n* = 56), and HTH-type transcriptional activator RhaR (*n* = 51). Of the positively associated gene clusters with sedentary controls, the most numerous annotations following hypothetical proteins included Sensor histidine kinase RcsC (*n* = 71), TonB-dependent receptor P3 (*n* = 66), Tyrosine recombinase XerC (*n* = 30), TonB-dependent receptor SusC (*n* = 27), and Vitamin B12 transporter BtuB (*n* = 27).

## DISCUSSION

In this study, we identify potentially exercise-associated features through bioinformatic analysis of all publicly available athlete gut metagenomic studies on rugby^[11]^, cyclists^[14]^, the Irish Olympic team^[21]^, cricketers^[21]^ and Boston Marathon runners^[12]^, and one currently unpublished study from our laboratory, (Irish runners). To establish a comparative framework, we used a sedentary control population as a reference [Figure 1].

Initially, we employed short-read taxonomic profiling to gain insights into microbial diversity, examining the distribution and abundance of microbial taxa among various athlete groups and the sedentary control population [Figures 2 and 3]. Following this, we complemented these findings with a MAG-based approach, which serves as the primary focus of this article. This integrated approach aimed to mitigate inconsistencies in results, which are attributed to methodological challenges inherent to metagenomic assembly pipelines. These challenges include higher coverage requirements and limitations stemming from sequence repeats and strain-level diversity, which are more pronounced in metagenomic assembly pipelines compared to short-read approaches^[24]^.

Our analysis unveiled discernible exercise-associated effects on microbial diversity, with perceivable differences within microbiome profiles of athletes compared to the sedentary control population [Figure 2B]. Such differences included statistically higher relative abundances of the phyla *Firmicutes* and *Actinobacteria* [Figure 2C], predominantly contributing to the observed differentially abundant features associated with the athlete population. These features were further evaluated using a MAG-based pipeline, where the corresponding MAG-encoded genes were systematically cataloged. Following the taxonomic assignment of MAGs [Figure 4], we found most unclassified detections (at the genus level or above) to be within the phylum *Firmicutes* [Figure 2C]. Our subsequent analysis revealed that several novel species of MAGs were recovered more than once based on pairwise calculations of two novel MAGs with the ANI score of ≥ 95%. The repeated identification of the same MAG taxon at the species level provides confidence in the MAG recovery pipeline and the novel isolate sequence. Future analysis should explore these novel MAGs further and assess their possible functions and safety within the gut microbiome.

Further analysis focused on several genera commonly elevated in athletes. Variations in species abundance among athlete populations were observed across the genera of interest, except for *Prevotella.* Notably, *Alistipes* accounted for a sizable proportion of differentially abundant features. We further observed significant increases in the relative abundance of *Alistipes*, *Faecalibacterium*, *Roseburia*, and *Ruminococcus* within the athlete population compared to the control sedentary population [Supplementary Figure 1]. These trends were consistent in the genera *Alistipes* and *Ruminococcus* when examining differences in MAG recovery within the athlete population compared to the control group [Supplementary Figure 1]. Lastly, we also recovered novel MAGs for the following genera: *Alistipes*, *Faecalibacterium*, *Prevotella*, and *Ruminococcus*.

These findings align with previous research associating these taxa with athlete populations^[10,11,13-17,19]^. *Prevotella* spp. have been previously correlated with muscle recovery due to their ability to synthesize branched-chain amino acids (BCAA)^[10]^; species of the genera *Bacteroides*, *Eubacterium*, *Roseburia*, and *Rumiococcus*^[40-42]^ have been shown to produce short chain fatty acids (SCFAs), and although the role of *Alistipes* in the gut remains largely unknown, it has been hypothesized that some species within the genus produce SCFAs^[43]^. Understanding the current controversial status of *Alistipes* spp. is crucial due to their implicated roles in various health conditions such as cancer^[44]^ and depression^[45]^, as well as their protective effects against colitis^[46]^ and cardiovascular issues^[47]^. These conflicting findings make it challenging to definitively ascertain their role in the athlete microbiome, as reviewed in^[43]^. With that being said, there is likely a strain/species difference that determines whether a particular *Alistipes* isolate is “good” or “bad”, and this dataset can possibly aid in answering that question by providing potential reference genomes, including those previously unclassified. Recent studies have highlighted the potential metabolic benefits of certain members of the *Collinsella* genus, which produce the SCFA butyrate^[48]^, an important metabolite involved in energy harvest, which is particularly relevant for athletes. *Faecalibacterium* spp., known for their immunomodulatory properties and SCFA production, may also play a vital role^[49,50]^.

In contrast, when considering the control sedentary populations, we observed statistically significant increases in the relative abundance and MAG recovery of *Proteobacteria* and *Bacteroidota* phyla [Figure 2C]. A higher relative abundance of *Bacteroides* spp. has also been previously noted in the original study by Barton *et al*.^[11]^. The results are also consistent with King *et al.*^[51]^, who estimated that *Bacteroidetes* typically represent 19.7% of the adult microbiome on average. Of course, it has to be noted that there were differences in sequencing depth [Figure 1C] influencing downstream analysis, such as MAG recovery, which could influence the reported differences observed between the different types of sport; however, the results of this paper are comparable to the shotgun analysis found in the original studies.

Overall, the species identified are not only in agreement with the original studies but also provide an expansion of our current knowledge of the sport-specific signatures present. Perhaps more studies should aim to expand our understanding of the relationship between shotgun and MAG analysis and whether those could be compared with accuracy. Additionally, the identified species have been associated with having a positive net impact on gut health and may play an important role in athlete health and performance. The results presented here warrant further investigation into these species and their potential role as next-generation probiotics.

Scoary facilitated studying the association of genes and their presence or absence with specific traits, revealing insights into the genetic underpinnings of athletic performance. Athletes exhibited gene clusters featuring Adaptive-response sensory-kinase SasA and Multidrug export protein MepA. SasA plays a pivotal role in sensing environmental cues and orchestrating cellular responses accordingly^[52]^. Given the rigorous training and stressors encountered by athletes, genes linked to adaptive responses like SasA could enhance their ability to cope with the physiological demands of intense physical activity and competition. MepA’s involvement in exporting various compounds from bacterial cells^[53]^ suggests its potential role in efficiently eliminating metabolic by-products or toxins generated during exercise, thereby promoting metabolic health and recovery. Moreover, the analysis uncovered additional gene clusters, albeit less prevalent, that could contribute to athletic performance. Notably, the enzyme fructose-bisphosphate aldolase (fba)^[54]^, which is involved in glycolysis and energy production, emerged as significant. Additionally, ilvC responsible for BCAA synthesis crucial for muscle development^[55]^ and pckA, implicated in glycogen metabolism and glucose synthesis^[56,57]^, were identified.

In conclusion, this catalog of MAGs and their subsequent genes is the most comprehensive to date in athletes. This MAG catalog has further enhanced our current understanding regarding the athlete gut microbiome and especially the phenomenon of sport-specific microbiome signatures. Our analysis also reveals differences in sport-associated gene clusters, offering new avenues for investigating the relationship between athletic performance and the gut microbiome. However, it is important to acknowledge the challenges inherent in shotgun metagenomic sequencing, including biases in sequencing chemistry^[58]^ that may result in incomplete representation of certain genomes, particularly those with cytosine (C) or guanine (G) rich regions (GC)-rich regions^[59,60]^.

Overall, the utility of applying shotgun metagenomics approaches to such data, in particular MAG recovery, by identifying sport-specific microbiome signatures, as well as recovering novel MAGs with potential applications as next-generation probiotics, could lead to a better understanding of the complex fitness-microbiome paradigm. The findings contained within this work can serve as a resource for future analysis to gain further insight into the field, as well as the possible role of these identified novel MAGs in the microbiome and their potential as next-generation probiotics.

## DECLARATIONS

### Availability of data and materials

All metagenomics data can be retrieved from NCBI SRA repository through their SRA Accession Number reported in Figure 1D. High-quality (HQ) MAGs assembled in this study have been deposited in the Open Science Framework (OSF) and can be accessed using the following DOI: 10.17605/OSF.IO/9DT6Y.

### Copyright

© The Author(s) 2024.
