# Supplementary material for: Cataloging metagenome-assembled genomes and microbial genes from the athlete gut microbiome
Source: Microbiome Res Rep. 2024 Jul 22;3(4):41. doi: 10.20517/mrr.2023.69 (PMC11684919; doi:10.20517/mrr.2023.69)
Supplement: Supplementary file 2 [file mrr-3-4-41-SupplementaryFigure.pdf]

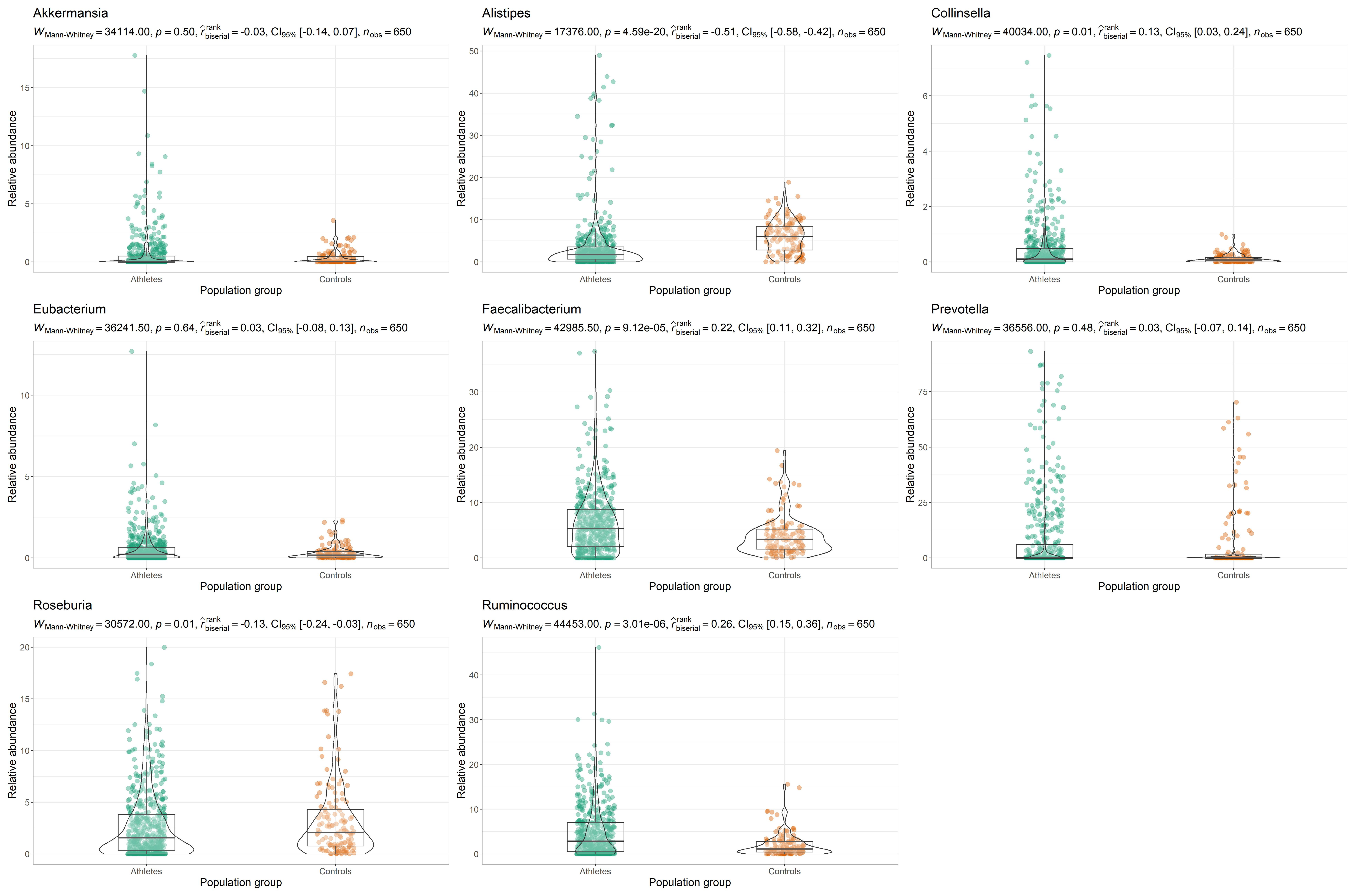

Akkermansia
$W_{Mann-Whitney}$ = 34114.00, p = 0.50, $\hat{r}_{biserial}^{rank}$ = -0.03, $CI_{95\%}$ [-0.14, 0.07], n_{obs} = 650
Alistipes
$W_{Mann-Whitney}$ = 17376.00, p = 4.59e-20, $\hat{r}_{biserial}^{rank}$ = -0.51, $CI_{95\%}$ [-0.58, -0.42], n_{obs} = 650
Collinsella
$W_{Mann-Whitney}$ = 40034.00, p = 0.01, $\hat{r}_{biserial}^{rank}$ = 0.13, $CI_{95\%}$ [0.03, 0.24], n_{obs} = 650
Eubacterium
$W_{Mann-Whitney}$ = 36241.50, p = 0.64, $\hat{r}_{biserial}^{rank}$ = 0.03, $CI_{95\%}$ [-0.08, 0.13], n_{obs} = 650
Faecalibacterium
$W_{Mann-Whitney}$ = 42985.50, p = 9.12e-05, $\hat{r}_{biserial}^{rank}$ = 0.22, $CI_{95\%}$ [0.11, 0.32], n_{obs} = 650
Prevotella
$W_{Mann-Whitney}$ = 36556.00, p = 0.48, $\hat{r}_{biserial}^{rank}$ = 0.03, $CI_{95\%}$ [-0.07, 0.14], n_{obs} = 650
Roseburia
$W_{Mann-Whitney}$ = 30572.00, p = 0.01, $\hat{r}_{biserial}^{rank}$ = -0.13, $CI_{95\%}$ [-0.24, -0.03], n_{obs} = 650
Ruminococcus
$W_{Mann-Whitney}$ = 44453.00, p = 3.01e-06, $\hat{r}_{biserial}^{rank}$ = 0.26, $CI_{95\%}$ [0.15, 0.36], n_{obs} = 650
Relative abundance
Population group
Athletes
Controls

Supplementary Figure 1. Relative abundance of genera detections often associated with the athlete population at the genus levels across “Athletes” (Green) and “Controls” populations (Orange). The bounds, whiskers, and percentile of each box plot represented maximum, 75th percentile, median, 25th percentile, and minimum from the top to the bottom, respectively.
